# Ongoing transmission of *Entamoeba histolytica* among newly diagnosed people living with HIV in Taiwan, 2009-2018

**DOI:** 10.1371/journal.pntd.0008400

**Published:** 2020-06-12

**Authors:** Sung-Hsi Huang, Mao-Song Tsai, Chun-Yuan Lee, Chin-Shiang Tsai, Chun-Eng Liu, Yuan-Ti Lee, Hong-An Chen, Ling-Ya Chen, Yu-Man Lu, Wan-Chen Tsai, Wei-Ting Hsu, Wang-Da Liu, Chia-Jui Yang, Hsin-Yun Sun, Wen-Chien Ko, Po-Liang Lu, Chien-Ching Hung, Taiwan HIV Study Group

**Affiliations:** 1 Department of Internal Medicine, National Taiwan University Hospital Hsin-Chu Branch, Hsin-Chu, Taiwan; 2 Department of Tropical Medicine and Parasitology, National Taiwan University College of Medicine, Taipei, Taiwan; 3 Department of Internal Medicine, Far Eastern Memorial Hospital, New Taipei City, Taiwan; 4 School of Medicine, College of Medicine, Fu Jen Catholic University, New Taipei City, Taiwan; 5 Department of Internal Medicine, Kaohsiung Medical University Hospital and College of Medicine, Kaohsiung Medical University, Kaohsiung, Taiwan; 6 Department of Internal Medicine, National Cheng Kung University Hospital, and College of Medicine, National Cheng Kung University, Tainan, Taiwan; 7 Department of Internal Medicine, Changhua Christian Hospital, Changhua, Taiwan; 8 School of Medicine, Chung Shan Medical University, Taichung, Taiwan; 9 Department of Internal Medicine, Chung Shan Medical University Hospital, Taichung, Taiwan; 10 Center of Infection Control, National Taiwan University Hospital, Taipei, Taiwan; 11 Department of Internal Medicine, National Taiwan University Hospital Biomedical Park Hospital, Hsin-Chu, Taiwan; 12 Department of Internal Medicine, National Taiwan University Hospital Yun-Lin Branch, Yun-Lin County, Taiwan; 13 Department of Internal Medicine, National Taiwan University Cancer Center, Taipei, Taiwan; 14 Department of Internal Medicine, National Taiwan University Hospital and National Taiwan University College of Medicine, Taipei, Taiwan; 15 Department of Medical Research, China Medical University Hospital; 16 China Medical University, Taichung, Taiwan; Hitit University, Faculty of Medicine, TURKEY

## Abstract

Recent outbreaks of enterically transmitted infections, including acute hepatitis A and shigellosis, have raised the concerns of increasing *Entamoeba histolytica* infection (EHI) among people living with HIV (PLWH) in Taiwan. This study investigated the prevalence of EHI, its temporal trends, and associated factors among newly diagnosed PLWH in Taiwan. Medical records of newly diagnosed PLWH at six medical centers in Taiwan between 2009 and 2018 were reviewed. The annual prevalence of invasive amoebiasis and seroprevalence of *E*. *histolytica* were determined and examined by the Cochran-Armitage test. The clinical characteristics associated with invasive amoebiasis and seropositivity for *E*. *histolytica* were analyzed in multivariable regression models. Among 5362 patients seeking HIV care at six medical centers in Taiwan during the 10-year study period, 119 (2.2%) had invasive amoebiasis at the time or within six months of their HIV diagnosis. Among 3499 who had indirect hemagglutination antibody (IHA) determined, 284 (8.1%) had positive IHA (≥1:32) and 205 (5.9%) had high-titre IHA (≥1:128). The prevalence of invasive amoebiasis increased from 1.3% in 2012 to 3.3% in 2018 (*p* = 0.024). Invasive amoebiasis was independently associated with a greater age, men who have sex with men, rapid plasma reagin titre ≥1:4, and concurrent shigellosis and giardiasis. Increasing prevalence of invasive amoebiasis among newly diagnosed PLWH in Taiwan calls for strategies to prevent ongoing transmission in this population. Routine screening of EHI for early diagnosis and treatment is recommended, especially among men who have sex with men and those who present with other sexually or enterically transmitted infections.

## Author summary

Outbreaks of enterically transmitted infection, including acute hepatitis A and shigellosis, among men who have sex with men and people living with HIV have been reported in Taiwan and in many developed countries in recent years. This study reveals that the prevalence of invasive amoebiasis among newly diagnosed people living with HIV increased in Taiwan since 2012, accompanied by increasing seroprevalence of syphilis and hepatitis A virus infection. This study also shows that concurrent infections with shigellosis and giardiasis and history of syphilis were independently associated with invasive amoebiasis, which indicates that transmission of *Entamoeba histolytica* might have occurred through sexual behaviours that increased faecal-oral contact in this population. In the era of improved access to HIV prevention and treatment, emerging and re-emerging enterically transmitted infections, including amoebiasis, pose an ongoing health threat to at-risk individuals and the public as a whole.

## Introduction

Amoebiasis, an infection caused by the protozoan *Entamoeba histolytica*, is the third leading cause of death from parasitic disease worldwide [1]. The majority of *E*. *histolytica* infection (EHI) are asymptomatic; however, invasive amoebiasis, most commonly in the form of colitis or liver abscess, occurs in 10% to 20% of infected individuals and can lead to mortality [1,2], morbidity and increased medical costs [3]. Traditionally, residents, immigrants and returned travelers from endemic countries where ingestion of mature amoebic cysts from contaminated food, water or hands occurs, as well as the individuals who are institutionalized are at the highest risk for EHI [4,5].

Since 1980s, amoebiasis has been recognised as an emerging sexually transmitted infection, especially among men who have sex with men (MSM) and those who are engaged in oral-anal sex [6–8]. Previous serological studies indicated that the seroprevalence of EHI among people living with HIV (PLWH) and those at high risk for HIV infection ranged from 1% to 21% [8] and high anti-*E*. *histolytica* antibody titres (e.g. indirect hemagglutination antibody (IHA) ≥1:128) were predictive of clinical infections [7]. With the characteristics of a low infectious dose, chlorine resistance, and stability in difficult environment for *E*. *histolytica*, EHI continues to pose a public health threat even in the developed countries [9]. Recent outbreaks in North America [10] and Europe [11] highlight its potential for transmission in countries where amoebiasis is not endemic.

Coinciding with the improvement of access to HIV treatment and pre- and post-exposure prophylaxis, increasing incidences of syphilis, chlamydia, gonorrhea, and other sexually transmitted infections among MSM have been observed in several high-income countries [12–14]. In Japan, a large hospital-based study demonstrated an increasing seroprevalence of EHI from 2004 to 2013 and the trend was observed in females and HIV-negative individuals as well as PLWH [15]. In Taiwan, recent outbreaks of shigellosis and acute hepatitis A between 2015 and 2017 disproportionately affected PLWH and MSM [16,17], suggesting that transmission of these enteric pathogens might have occurred through sexual behaviours that increased faecal-oral contacts. The potential for *E*. *histolytica* to spread through the same route raised concerns [10]. In this study, we aimed to investigate the temporal changes of the prevalence of invasive amoebiasis and anti-*E*. *histolytica* positivity among newly diagnosed PLWH in Taiwan from 2009 to 2018 and to identify its associated factors.

## Methods

### Ethics statement

The study was approved by the Research Ethics Committees of National Taiwan University Hospital [201003112R] and Far Eastern Memorial Hospital [105040-F] and Institutional Review Boards of Chung Shan Medical University Hospital [CS14034], Changhua Christian Hospital [160408], Kaohsiung Medical University Hospital [KMUHIRB-SV(I)-20160043], and National Cheng Kung University Hospital [B-BR-105-038]. The informed consent was waived. The study was conducted according to the principles expressed in the Declaration of Helsinki.

### Study setting and patients

In this cross-sectional multicentre study, we reviewed the medical records of all PLWH who presented to six major medical centers in Taiwan for the first time between 2009 and 2018. We included patients with newly diagnosed HIV infection; and those who had been diagnosed at other hospitals before presenting to the six participating hospitals or had been diagnosed before 2009 were excluded. Available clinical and laboratory data at the time of HIV diagnosis and within six months before and after HIV diagnosis were collected. Those who died or were lost to follow-up shortly after the HIV diagnosis, those who refused testing, and those who did not complete baseline investigations for other reasons were excluded.

Five of the six participating hospitals are located in the metropolitan cities (Taipei City, New Taipei City, Taichung City, Tainan City, and Kaohsiung City) and one in Changhua County. According to the Taiwan Centers for Disease Control (TCDC), 15,938 incident HIV cases were reported from these administrative areas in the 10-year study period, accounting for 75% of all incident HIV cases across Taiwan in the same period [18].

Routine baseline laboratory investigations for newly diagnosed PLWH in Taiwan included CD4 lymphocyte count, plasma HIV RNA load, complete blood cell count, liver- and renal-function tests, blood glucose, lipid profile, and serology for hepatitis A virus (HAV), hepatitis B virus (HBV), hepatitis C virus (HCV), and syphilis according to the national HIV treatment guidelines [19,20]. Considering a higher prevalence of EHI among PLWH, measurement of anti-*E*. *histolytica* antibody, although not mandatory, was also recommended in the guidelines [19,20]. All of the laboratory investigations at baseline or during follow-up regardless of initiation of antiretroviral therapy are reimbursed by the special budget of TCDC and the National Health Insurance.

### Definitions of study outcomes

Prevalences of invasive amoebiasis and seropositivity for *E*. *histolytica* were the outcomes of interest in this study. We defined invasive amoebiasis, including amoebic liver abscess and/or colitis, as having (1) at least one related symptom (including diarrhea, bloody stools, abdominal pain, nausea, vomiting, or fevers); liver abscess on imaging; or colitis by colonoscopy; (2) at least one positive parasitological test (including test for specific amoebic antigen, nucleic-acid amplification, and serology) or pathologic finding; and (3) treatment with metronidazole, with or without paromomycin or iodoquinol.

Anti-*E*. *histolytica* antibody was determined by IHA assay. Negative IHA was defined by an IHA titre <1:32, low-titre IHA by a titre of 1:32 or 1:64, and high-titre IHA by a titre of ≥1:128 [21,22].

### Laboratory investigations

During the study period, determinations of plasma HIV RNA load, CD4 lymphocyte count, and serological tests for syphilis and viral hepatitis (HAV, HBV and HCV) were performed using certified commercial tests at each participating hospital. Because of the acute hepatitis A outbreak in Taiwan between 2015 and 2017, an HAV vaccination campaign and program among PLWH were carried out in Taiwan [16] and serum anti-HAV immunoglobulin G (IgG) levels were measured routinely before HAV vaccination. Pre-vaccination serum anti-HAV IgG levels were used when recording anti-HAV antibody for each individual. IHA assay (Cellognostics; Boehringer Diagnostics, Marburg, Germany) that quantitatively detects specific antibodies to *E*. *histolytica* was performed by following the manufacturer’s instructions in the routine laboratory of each hospital [23].

### Statistical analysis

Statistical analyses were performed using the R statistics software (version 3.3.3). The annual prevalences of IHA seropositivity, high-titre IHA seropositivity, and invasive amoebiasis between 2009 and 2018 were calculated and the trends were examined by Cochran-Armitage test. Non-categorical variables were compared using Student’s t-test or Mann-Whitney U test and categorical variables were compared using chi-square test or Fisher’s exact test between the patients with and those without invasive amoebiasis. Clinical variables with *p*-value <0.05 were entered into multivariable general linear regression models with backward selection and missing values were treated by exclusion to identify independent factors associated with invasive amoebiasis. The same procedure was repeated to identify factors associated with high-titre IHA. The variables with *p*-value <0.05 were deemed statistically significant throughout the analyses.

## Results

### Characteristics of the patients

**Fig 1:**
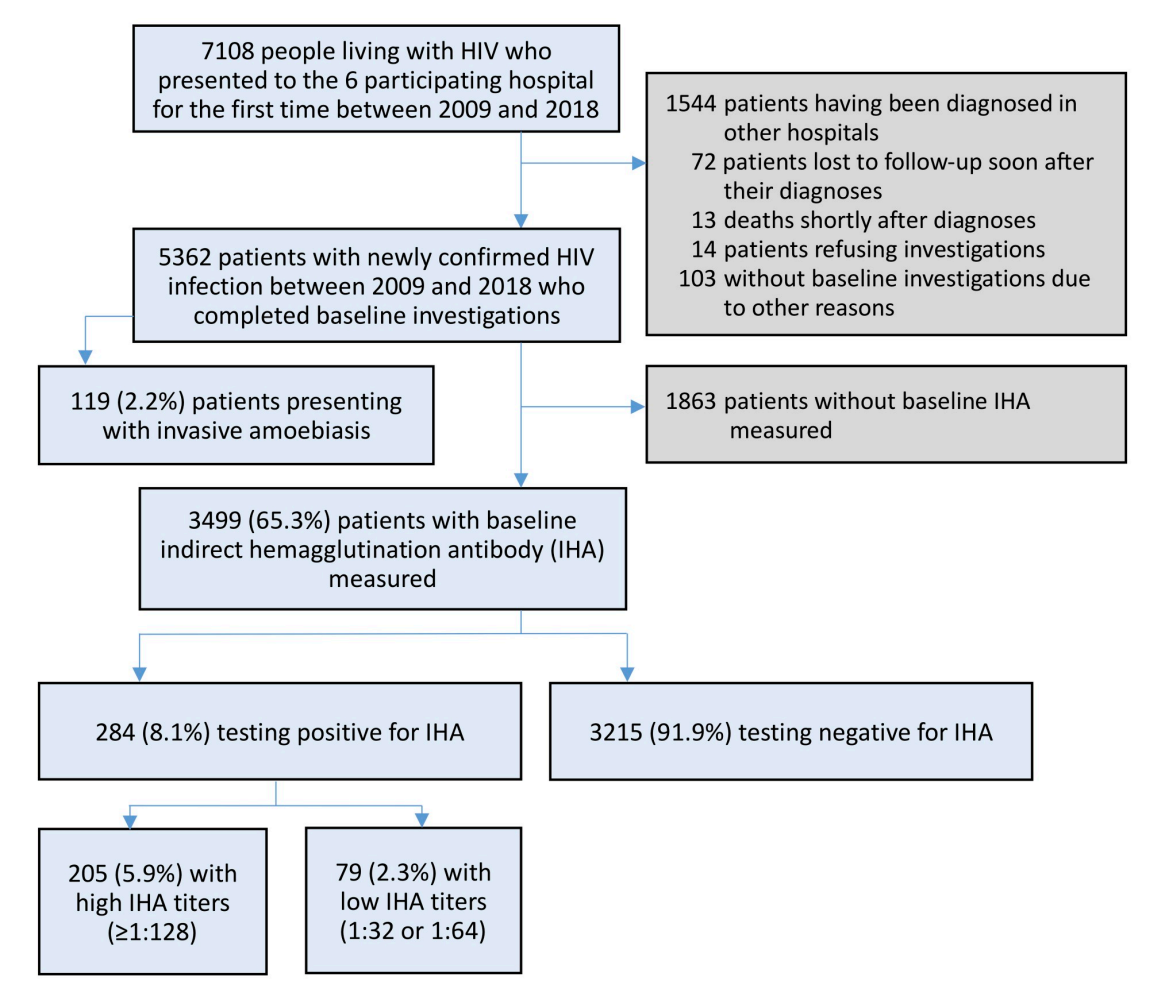
Study flow.

During the 10-year study period, 7108 PLWH seeking care at the six participating hospitals for the first time were screened for inclusion, and 5362 who had newly diagnosed HIV infection and completed baseline evaluation were included for analysis (Fig 1), which accounted for 25.1% of 21321 newly diagnosed PLWH in Taiwan from 2009 to 2018. The clinical characteristics of the cohort are shown in Table 1. The majority of the included patients were MSM (84.9%) in the age group of 20 to 39 years (78.9%). Late presenters for HIV care were still commonly encountered, with 34.6% presenting with a CD4 lymphocyte count <200 cells/μl, 61.5% <350 cells/μl, and 16.5% having at least one opportunistic infection. At time of HIV diagnosis, 20.0% had a rapid plasma reagin (RPR) titre of ≥1:4.

**Table 1:** Comparisons of characteristics between newly diagnosed people living with HIV who had and those who did not have invasive amoebiasis.

	Total	With invasive amoebiasis	Without invasive amoebiasis	Univariable analysis
	N = 5362	N = 119	N = 5243	*p*-value
Age, median (IQR), years	29.3 (24.5, 36.0)	33.8 (27.6, 39.4)	29.2 (24.4, 36.0)	**<0.001**
<20, no. (%)	222 (4.1)	0 (0.0)	222 (4.2)	**<0.001**
20–29	2633 (49.1)	38 (31.9)	2595 (49.5)	
30–39	1600 (29.8)	54 (45.4)	1546 (29.5)	
40–49	630 (11.7)	20 (16.8)	610 (11.6)	
≥50	277 (5.2)	7 (5.9)	270 (5.1)	
Male, no. (%)	5219 (97.3)	119 (100.0)	5100 (97.3)	0.081
Mode of transmission, no. (%)				
Men who have sex with men	4555 (84.9)	115 (96.6)	4440 (84.7)	**<0.001**
People who inject drugs	232 (4.3)	2 (1.7)	230 (4.4)	0.247
Location of residence, no. (%)				
Northern Taiwan	2744 (51.2)	76 (63.9)	2668 (50.9)	**0.023**
Central Taiwan	796 (14.8)	12 (10.1)	784 (15.0)	
Southern Taiwan	1822 (34.0)	31 (26.1)	1791 (34.2)	
Coinfection, no. (%)				
HBsAg-positive [N = 5258]	527 (10.0)	15 (12.7)	512 (10.0)	0.350
Anti-HCV-positive [N = 5296]	418 (7.9)	9 (7.7)	409 (7.9)	>0.999
RPR titre ≥1:4, no. (%) [N = 5308]	1063 (20.0)	39 (33.3)	1024 (19.7)	**0.001**
Anti-HAV IgG-positive, no. (%) [N = 4601]	795 (17.3)	22 (22.4)	773 (17.2)	0.177
Enterically transmitted infections, no. (%)	124 (2.3)	10 (8.4)	114 (2.2)	**<0.001**
Shigellosis	4 (0.1)	3 (2.5)	1 (0.02)	**<0.001**
Salmonellosis	72 (1.3)	4 (3.4)	68 (1.3)	0.075
Giardiasis	14 (0.3)	4 (3.4)	10 (0.2)	**<0.001**
Cryptosporidiosis	11 (0.2)	1 (0.8)	10 (0.2)	0.219
Acute hepatitis A	28 (0.5)	0 (0.0)	28 (0.5)	>0.999
Any opportunistic infection, no. (%)	886 (16.5)	29 (24.4)	857 (16.3)	**0.024**
Pneumocystosis	597 (11.1)	16 (13.4)	581 (11.1)	0.379
Tuberculosis	80 (1.5)	0 (0.0)	80 (1.5)	0.425
Cryptococcosis	67 (1.2)	3 (2.5)	64 (1.2)	0.186
White blood cell count, median (IQR), x 10^3^ cells/μl [N = 5353]	5.6 (4.4, 7.0)	5.7 (4.3, 8.2)	5.6 (4.4, 7.0)	0.230
Hemoglobin, median (IQR), g/dL [N = 5338]	14.1 (12.6, 15.1)	12.6 (10.5, 14.2)	14.1 (12.7, 15.1)	**<0.001**
Any abnormal liver function test result, no. (%)	1614 (30.1)	43 (36.1)	1571 (30.0)	0.157
Plasma HIV RNA load, median (IQR), log_10_ copies/ml [N = 5324]	4.8 (4.3, 5.3)	5.0 (4.6, 5.5)	4.8 (4.3, 5.3)	**0.001**
HIV RNA load >5 log_10_ copies/ml, no. (%)	2154 (40.5)	67 (56.3)	2087 (40.1)	**<0.001**
CD4 lymphocyte count, median (IQR), cells/μl [N = 5355]	289 (133, 441)	226 (75, 349)	290 (133, 443)	**0.002**
CD4 <200, n (%)	1853 (34.6)	55 (46.2)	1798 (34.3)	**0.012**
CD4 200–350	1438 (26.9)	34 (28.6)	1404 (26.8)	
CD4 350–500	1053 (19.7)	15 (12.6)	1038 (19.8)	
CD4 ≥500	1011 (18.9)	15 (12.6)	996 (19.0)	
Diarrhea, no. (%)	559 (10.4)	103 (86.6)	456 (8.7)	**<0.001**

Boldface indicates a statistically significant result.

Abbreviations: HAV, hepatitis A virus; HBsAg, hepatitis B virus surface antigen; HCV, hepatitis C virus; IQR, interquartile range; RPR, rapid plasma reagin

### Prevalence of invasive amoebiasis and its associated factors

Overall, invasive amoebiasis was diagnosed in 119 patients (2.2%) around the time of their HIV diagnosis (Fig 1). Among the patients who had invasive amoebiasis, 35 (29.4%) had amoebic liver abscess, and 72 (60.5%) required hospitalization. Three patients (2.5%), who presented with advanced immunocompromised state (CD4 count, 0, 21, and 57 cells/μl, respectively) and other serious opportunistic infections (1 pneumocystosis, 1 CMV colitis, and 1 both), died within three months of the HIV diagnosis.

In the multivariable analysis, factors independently associated with invasive amoebiasis were age (adjusted odds ratio [aOR], per 1-year increase, 1.001, 95% confidence interval [CI] 1.001–1.002), MSM (aOR 1.027, 95% CI 1.015–1.040), RPR titre of ≥1:4 (aOR 1.016, 95% CI 1.006–1.026), and concurrent shigellosis (aOR 1.757, 95% CI 1.488–2.076) and giardiasis (aOR 1.233, 95% CI 1.141–1.332) (Table 2).

**Table 2:** Multivariable analysis of factors associated with invasive amoebiasis.

	Adjusted odds ratio	*p*-value
Age, per 1-year increase	1.001 (1.001–1.002)	**<0.001**
Men who have sex with men	1.027 (1.015–1.040)	**<0.001**
Rapid plasma reagin titre ≥1:4	1.016 (1.006–1.026)	**0.001**
Shigellosis	1.757 (1.488–2.076)	**<0.001**
Giardiasis	1.233 (1.141–1.332)	**<0.001**

Variables entered in the multivariable analysis include age, being men who have sex with men, geographic locations, rapid plasma reagin titre ≥1:4, baseline CD4 lymphocyte count, baseline plasma HIA RNA load, shigellosis, giardiasis, and any opportunistic infection.

### Seroprevalence of *E*. *histolytica* infection

IHA testing was performed in 50% to 65% of newly diagnosed PLWH before 2014 and in 72% to 79% after 2015 (S1 Fig). Of the 3499 (65.3% of all included patients) who had IHA determined at baseline, 284 (8.1%) had positive IHA and 205 (5.9%) had high-titre IHA (Fig 1). A lower estimate of the prevalence of positive IHA and high-titre IHA would be 5.3% and 3.8%, respectively, if the 1863 patients who were not tested by IHA assays were all presumed to be seronegative for *E*. *histolytica*. Ninety-one (44.4%) of 205 patients who had high-titre IHA, 13 (16.5%) of 79 who had low-titre IHA, and 15 (0.5%) of 3215 who had negative IHA received a diagnosis of invasive amoebiasis within six months of their HIV diagnosis. Compared with the patients who did not have IHA tests, those who had IHA determined were younger, less likely to be injecting drug users, and more likely to have diarrhea or anaemia after adjustments for geographic locations and year of HIV diagnosis (S1 Table).

### Factors associated with high-titre IHA

**Fig 2:**
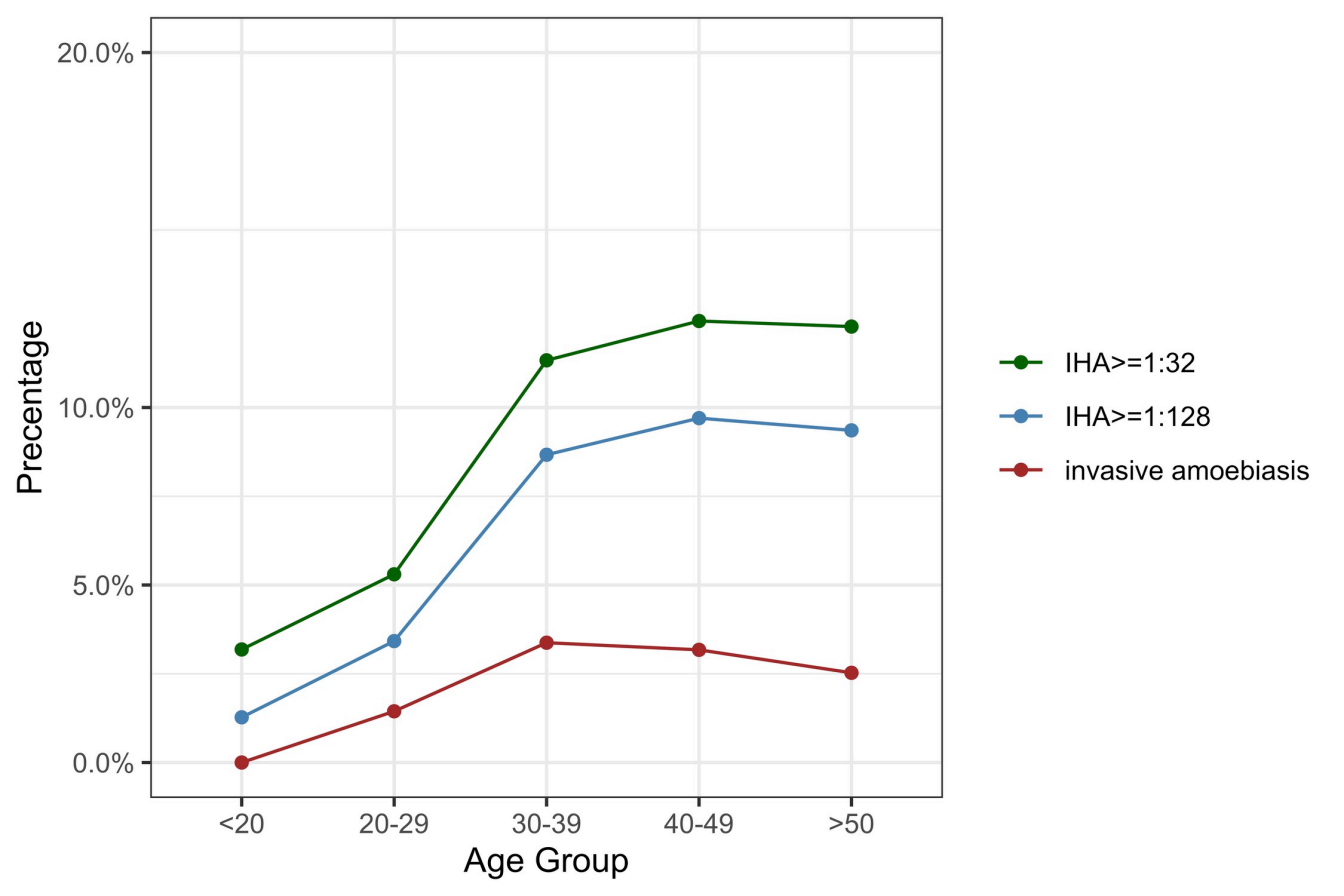
Prevalence of *Entamoeba histolytica* infection among newly diagnosed people living with HIV according to age group.

Similar factors that were associated with invasive amoebiasis, including age, MSM, RPR titre ≥1:4, and shigellosis, were identified to be independently associated with high-titre IHA among those who had IHA determined at baseline (S2 and S3 Tables). Patients who presented with acute hepatitis A and cryptococcosis at the time of their HIV diagnosis were also more likely to have high-titre IHA (aOR 1.121 [95% CI 1.013–1.241] and 1.084 [95% CI 1.011–1.16], respectively). The prevalence of positive IHA, high-titre IHA, and invasive amoebiasis increased with age and plateaued before the age of 40 years (Fig 2). Neither invasive amoebiasis nor high-titre IHA was associated with CD4 lymphocyte counts at baseline.

### Trends of the annual prevalences of invasive amoebiasis, positive IHA, and high-titre IHA

**Fig 3:**
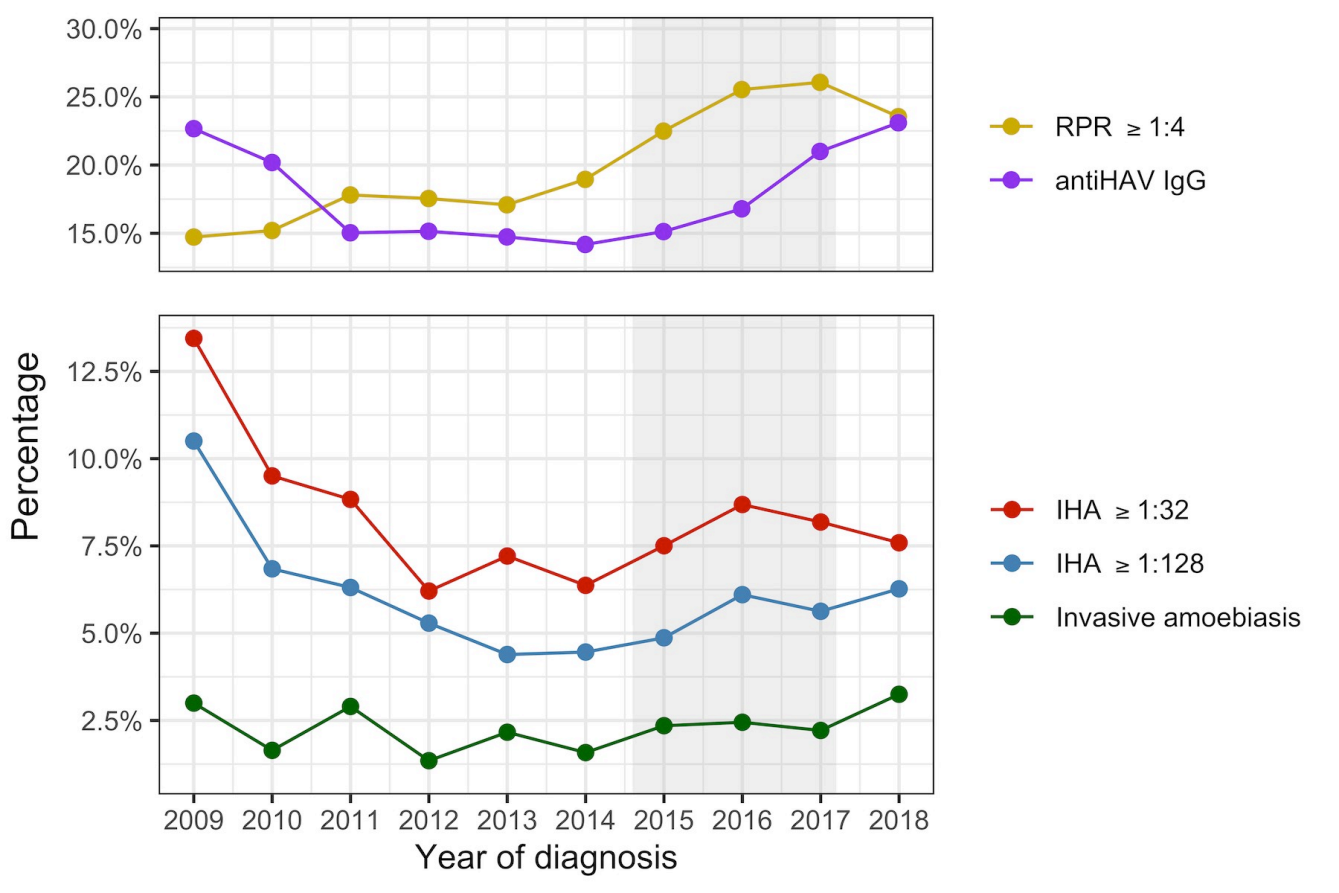
Trends of prevalence of invasive amoebiasis and seroprevalences of *Entamoeba histolytica*, hepatitis A virus (HAV), and syphilis among newly diagnosed people living with HIV from 2009 to 2018. The grey shade indicates the outbreak of acute hepatitis A in Taiwan from mid-2015 to 2017. The prevalence of invasive amoebiasis was calculated from all newly diagnosed people living with HIV, while the seroprevalences of amoebiasis, HAV and syphilis were calculated from 3499, 4601, and 5308 patients with indirect hemagglutination assay, anti-HAV immunoglobin G (IgG), and rapid plasma reagin (RPR) determined at baseline, respectively. An HAV vaccination campaign was started since late-2015. Serum anti-HAV IgG levels were measured routinely before vaccination and pre-vaccination anti-HAV IgG measurements were used in the analysis.

As shown in Fig 3, the annual prevalence of invasive amoebiasis decreased from 3.0% in 2009 to 1.3% in 2012 (p = 0.090), which then increased again to 3.3% in 2018 (p = 0.024). The annual prevalence of positive IHA and that of high-titre IHA demonstrated a similar trend with declines from 13.4% and 10.5% in 2009 to 6.2% and 5.3% in 2012, respectively, (p = 0.008 and 0.001, respectively), followed by increases to 7.6% and 6.3% in 2018, respectively (p = 0.104 and 0.151, respectively). The age distribution of the included patients did not change significantly throughout the study period (S2 Fig), while the proportion of the patients with CD4 lymphocyte counts less than 200 cells/μL and concurrent opportunistic infections decreased with time (p = 0.043 and 0.024, respectively; S3 and S4 Figs). When compared with the seroepidemiology of HAV and syphilis, the temporal changes of prevalence of invasive amoebiasis and seroprevalences of positive and high-titre IHA evolved in the same direction with the seroprevalence of HAV throughout the study period and that of syphilis after 2012 (Fig 3).

## Discussion

In this cross-sectional study, we investigated the prevalence of EHI among newly diagnosed PLWH at six medical centers in Taiwan from 2009 to 2018. We found that one out of every 45 newly diagnosed PLWH had concurrent invasive amoebiasis and one out of every 13 had positive IHA. There was a significant increase of invasive amoebiasis from 2012 to 2018. Being older, MSM, having RPR titres of ≥1:4, shigellosis, and giardiasis were independently associated with invasive amoebiasis.

Several outbreaks of enterically transmitted infections, including acute hepatitis A and shigellosis, among MSM and PLWH in Taiwan have been reported since 2015 [17,24]. Our study showed a trend of increasing prevalence of invasive amoebiasis from 2012 to 2018, which paralleled the trends of the seroprevalence of HAV and syphilis, as shown in Fig 3. Unlike shigellosis and HAV infection that commonly present with acute illnesses, amoebiasis is more likely to be asymptomatic or mild and overlooked [9]. The change of annual prevalence of invasive amoebiasis should raise concerns, especially when increase in confirmed cases of amoebiasis, a notifiable disease in Taiwan, was also observed from 2012 to 2018 in the national infectious disease statistics (S5 Fig) [18]. This indicates the ongoing, or even increasing, transmission of *E*. *histolytica* might be occurring not only among PLWH, but potentially also in the general population. Another potential explanation would be the introduction of a hypervirulent strain within the population [10]. As shown in this study, a significant proportion of these infections led to liver abscesses and hospitalizations. The need to enhance surveillance of enterically transmitted infections, including amoebiasis, among the at-risk populations could not be overemphasized.

Invasive amoebiasis and high-titre *E*. *histolytica* seropositivity were associated with several independent factors in this study. Among those, the association with seropositivity of *T*. *pallidum* infection and being MSM has been reported previously [4,25,26]. The finding that patients with concurrent shigellosis or giardiasis had higher risk for amoebiasis, however, has not been demonstrated before. Nevertheless, these findings are not unexpected, as the transmission of these infections requires low infectious doses and probably involves the same sexual practices [11,27,28]. In an outbreak of intestinal amoebiasis in Spain, two out of eight patients had concurrent shigellosis [11]. In two epidemiologic investigations from Canada and the United Kingdom, a higher male-to-female ratio of amoebiasis, shigellosis, and giardiasis among individuals of sexually active age groups were observed [29,30]. In our study, patients with invasive amoebiasis were more likely to have concurrent enterically transmitted infections (8.4% vs 2.2%). To extrapolate from the above findings, an enterically transmitted infection in MSM or PLWH should probably prompt clinicians to think about amoebiasis and other potential coinfections. Nowadays, the use of drugs for sex (chemsex) has become more common [31], and it has been demonstrated to be associated with outbreaks of shigellosis [17]. Whether or not this would also boost the spreading of other enterically transmitted infections, such as amoebiasis, warrants further investigation.

Diagnosis of EHI can be challenging in real-world settings. Patients identified to have invasive amoebiasis in this study were those who presented with active symptoms and cared by physicians who were vigilant to make the diagnosis. However, more patients were expected to have asymptomatic or mild EHI and could continue to transmit the infection, unless routine screening for EHI with accurate diagnostic tests could be implemented. The best tools for surveillance of amoebiasis remain to be determined. Serologic tests are relatively simple to perform and appear cost-effective for screening of amoebiasis [32]; however, even among symptomatic patients false-negative results can be observed in up to 38% [33,34], which means even with routine measurement of anti-*E*. *histolytica* antibodies, a substantial proportion of patients with EHI may remain undiagnosed. Moreover, in endemic areas like Taiwan, because a positive serological result cannot differentiate current and past infections, a confirmatory test is usually needed and the sequential testing strategy could be time-consuming. Stool amoebic antigen detection is available commercially, easy to perform, and has a rapid turnaround time; however, it requires fresh, un-frozen stool samples, and, moreover, its sensitivity varies widely [9,35]. Identification of *E*. *histolytica*-specific nucleic acids by polymerase-chain reaction (PCR) assays is highly sensitive and has become the gold standard in diagnosing EHI [35]. Routine testing by molecular tests to detect and treat EHI early in the high-risk populations may be an effective strategy to prevent potential outbreaks and to reverse the increasing trend. Further cost-effectiveness or cost-utility analyses are warranted to inform public health policy.

The study has several limitations. First, as mentioned above, the true prevalence of EHI may require routine screening by more accurate diagnostic tools, such as PCR assays. The criteria for invasive amoebiasis used in this study identified only symptomatic patients and under-represent the true prevalence of EHI. Second, a third of patients included in this study did not have IHA determined at baseline and the estimate of seroprevalence may be biased by the selection for testing. The seroprevalence of EHI in this study could be an overestimate since patients who had higher hemoglobin levels and who did not present with diarrhea were less likely to be tested by IHA (S1 Table). Proportion of patients who underwent IHA testing was higher after 2015. This coincided with the outbreaks of HAV and shigellosis and could be due to increased clinical vigilance regarding diarrheal diseases in the outbreak settings. The smaller proportion of patients who underwent IHA testing before 2014 may result in a higher estimate of IHA seroprevalence before 2014 and could potentially dampen the trend observed.

Although recent development of vaccines against *E*. *histolytica* in rodents and non-human primate models shows some promise [36,37], effective human vaccines to prevent EHI remain unavailable to date. In the developed countries where access to water safety, hygiene and sanitation are readily available, person-to-person transmission plays an important role in spreading of the pathogen. Thus, the appropriate strategies to prevent the transmission of EHI will rely upon active identification and timely initiation of appropriate treatment of the infected individuals, especially among the at-risk populations.

In conclusion, increasing invasive amoebiasis has been observed since 2012 along with other sexually transmitted and enterically transmitted infections among newly diagnosed PLWH in Taiwan. To identify and treat early, and to prevent potential outbreaks, routine investigations for amoebiasis among PLHW is indicated, especially among those who are MSM and those who present with other sexually transmitted and enterically transmitted infections.

## Supporting information

S1 Table Comparisons of characteristics between newly diagnosed people living with HIV who had indirect hemagglutination (IHA) assay performed at baseline and those who did not. (PDF)

S2 Table Comparisons of characteristics between newly diagnosed people living with HIV with and those without high indirect hemagglutination (IHA) titres (≥1:128) in univariable analyses. (PDF)

S3 Table Factors associated with a high indirect hemagglutination (IHA) titre (≥1:128) in multivariable analysis. (PDF)

S1 Fig Proportion of newly diagnosed people living with HIV who underwent indirect hemagglutination (IHA) testing between 2009 and 2018. (PDF)

S2 Fig Correlation between age and year of diagnosis among newly diagnosed people living with HIV in a scatter plot. (PDF)

S3 Fig Proportion of newly diagnosed people living with HIV in different CD4 lymphocyte count categories between 2009 and 2018. (PDF)

S4 Fig Proportion of newly diagnosed people living with HIV who presented with opportunistic infection at baseline between 2009 and 2018. (PDF)

S5 Fig Annual number of indigenous cases of confirmed amoebiasis reported to the Taiwan Centers for Disease Control between 2009 and 2018. Criteria for confirmed amoebiasis by Taiwan Centers for Disease Control included (1) positive *Entamoeba histolytica* nucleic-acid amplification test from any clinical specimens (including stool, tissue, and aspirate); (2) fever or right upper quadrant pain plus identification of amoebic trophozoites from the tissues; or (3) fever or right upper quadrant pain plus radiographic evidence of liver abscess and positive anti-*E*. *histolytica* antibody.
(PDF)

## References

[1] Lozano R, Naghavi M, Foreman K, Lim S, Shibuya K, Aboyans V, et al Global and regional mortality from 235 causes of death for 20 age groups in 1990 and 2010: a systematic analysis for the Global Burden of Disease Study 2010. Lancet. 2012;380: 2095–2128. 10.1016/S0140-6736(12)61728-0 23245604 PMC10790329

[2] Shirley D-A, Moonah S. Fulminant Amebic Colitis after Corticosteroid Therapy: A Systematic Review. PLoS Negl Trop Dis. 2016;10: e0004879–13. 10.1371/journal.pntd.0004879 27467600 PMC4965027

[3] Congly SE, Shaheen AAM, Meddings L, Kaplan GG, Myers RP. Amoebic liver abscess in USA: a population-based study of incidence, temporal trends and mortality. Liver Int. 2011;31: 1191–1198. 10.1111/j.1478-3231.2011.02562.x 21745303

[4] Lo Y-C, Ji D-D, Hung C-C. Prevalent and incident HIV diagnoses among Entamoeba histolytica-infected adult males: a changing epidemiology associated with sexual transmission—Taiwan, 2006–2013. PLoS Negl Trop Dis. 2014;8: e3222 10.1371/journal.pntd.0003222 25299178 PMC4191956

[5] Swaminathan A, Torresi J, Schlagenhauf P, Thursky K, Wilder-Smith A, Connor BA, et al A global study of pathogens and host risk factors associated with infectious gastrointestinal disease in returned international travellers. J Infect. 2009;59: 19–27. 10.1016/j.jinf.2009.05.008 19552961

[6] Burnham WR, Reeve RS, Finch RG. Entamoeba histolytica infection in male homosexuals. Gut. 1980;21: 1097–1099. 10.1136/gut.21.12.1097 7461471 PMC1419409

[7] Hung C-C, Chen PJ, Hsieh SM, Wong JM, Fang C-T, Chang SC, et al Invasive amoebiasis: an emerging parasitic disease in patients infected with HIV in an area endemic for amoebic infection. AIDS. 1999;13: 2421–2428. 10.1097/00002030-199912030-00014 10597784

[8] Hung C-C, Chang S-Y, Ji D-D. Entamoeba histolytica infection in men who have sex with men. The Lancet Infectious Diseases. 2012;12: 729–736. 10.1016/S1473-3099(12)70147-0 22917103

[9] Shirley D-AT, Farr L, Watanabe K, Moonah S. A Review of the Global Burden, New Diagnostics, and Current Therapeutics for Amebiasis. Open Forum Infect Dis. 2018;5: ofy161 10.1093/ofid/ofy161 30046644 PMC6055529

[10] Salit IE, Khairnar K, Gough K, Pillai DR. A possible cluster of sexually transmitted Entamoeba histolytica: genetic analysis of a highly virulent strain. Clin Infect Dis. 2009;49: 346–353. 10.1086/600298 19580413

[11] Escolà-Vergé L, Arando M, Vall M, Rovira R, Espasa M, Sulleiro E, et al Outbreak of intestinal amoebiasis among men who have sex with men, Barcelona (Spain), October 2016 and January 2017. Euro Surveill. 2017;22: 729 10.2807/1560-7917.ES.2017.22.30.30581 28797327 PMC5553055

[12] Unemo M, Bradshaw CS, Hocking JS, de Vries HJC, Francis SC, Mabey D, et al Sexually transmitted infections: challenges ahead. The Lancet Infectious Diseases. 2017;17: e235–e279. 10.1016/S1473-3099(17)30310-9 28701272

[13] Ndumbi P, Freidl GS, Williams CJ, Mårdh O, Varela C, Avellón A, et al Hepatitis A outbreak disproportionately affecting men who have sex with men (MSM) in the European Union and European Economic Area, June 2016 to May 2017. Euro Surveill. 2018;23: 85 10.2807/1560-7917.ES.2018.23.33.1700641 30131095 PMC6205254

[14] Yoshimura Y, Horiuchi H, Sawaki K, Miyata N, Kumazaki M, Usuku S, et al Hepatitis A outbreak among men who have sex with men, Yokohama, Japan, January to May 2018. Sex Transm Dis. 2018 10.1097/OLQ.0000000000000937 30395105

[15] Gatanaga H, Watanabe K, Kikuchi Y, Teruya K, Nagata N, Yanagawa Y, et al Increases in Entamoeba histolytica Antibody–Positive Rates in Human Immunodeficiency Virus–Infected and Noninfected Patients in Japan: A 10-Year Hospital-Based Study of 3,514 Patients. Am J Trop Med Hyg. 2016;95: 604–609. 10.4269/ajtmh.16-0134 27296390 PMC5014266

[16] Chen G-J, Lin K-Y, Hung C-C, Chang S-C. Hepatitis A Outbreak Among Men Who Have Sex With Men in a Country of Low Endemicity of Hepatitis A Infection. J Infect Dis. 2017;215: 1339–1340. 10.1093/infdis/jix123 28329351

[17] Wu H-H, Shen Y-T, Chiou C-S, Fang C-T, Lo Y-C. Shigellosis outbreak among MSM living with HIV: a case-control study in Taiwan, 2015–2016. Sex Transm Infect. 2019;95: 67–70. 10.1136/sextrans-2017-053410 29535222

[18] Taiwan Centers for Disease Control. Taiwan National Infectious Diseas Statistics System website. [cited 28 Feb 2020]. Available from: http://nidss.cdc.gov.tw/en/NIDSS_DiseaseMap.aspx

[19] Taiwan Centers for Disease Control. Guidelines for diagnosis and treatment of HIV/AIDS, 3rd edition. 2010. pp. 1–208. Available from: https://www.cdc.gov.tw/En/File/Get/3IyEeblAnfuaILiLst39kA

[20] Taiwan AIDS Society. Guidelines for diagnosis and treatment of HIV/AIDS, 5th edition. 2018. Available from: http://www.aids-care.org.tw/journal/treatment.asp.

[21] Hung C-C, Ji D-D, Sun HY, Lee Y-T, Hsu S-Y, Chang S-Y, et al Increased risk for Entamoeba histolytica infection and invasive amebiasis in HIV seropositive men who have sex with men in Taiwan. PLoS Negl Trop Dis. 2008;2: e175 10.1371/journal.pntd.0000175 18301730 PMC2254204

[22] Tsai J-J, Sun HY, Ke L-Y, Tsai K-S, Chang S-Y, Hsieh S-M, et al Higher seroprevalence of Entamoeba histolytica infection is associated with human immunodeficiency virus type 1 infection in Taiwan. Am J Trop Med Hyg. 2006;74: 1016–1019. 16760513

[23] Cellognost-Amoebiasis Package Insert. 2004 [cited 13 Oct 2019]. Available from: https://info.fda.gov.tw/MLMS/H0001D3.aspx?LicId=06014815

[24] Lin K-Y, Sun HY, Chen Y-H, Lo Y-C, Hsieh S-M, Sheng W-H, et al Effect of a Hepatitis A Vaccination Campaign During a Hepatitis A Outbreak in Taiwan, 2015–2017: A Modeling Study. Clinical Infectious Diseases. 2019;23: 3589–8. 10.1093/cid/ciz471 31157857

[25] Nagata N, Shimbo T, Akiyama J, Nakashima R, Nishimura S, Yada T, et al Risk factors for intestinal invasive amebiasis in Japan, 2003–2009. Emerging Infectious Diseases. 2012;18: 717–724. 10.3201/eid1805.111275 22515839 PMC3358059

[26] Watanabe K, Gatanaga H, Escueta-de Cadiz A, Tanuma J, Nozaki T, Oka S. Amebiasis in HIV-1-infected Japanese men: clinical features and response to therapy. PLoS Negl Trop Dis. 2011;5: e1318 10.1371/journal.pntd.0001318 21931875 PMC3172195

[27] Furness BW, Beach MJ, Roberts JM. Giardiasis surveillance—United States, 1992–1997. MMWR. 2000;49: 1–13. 10955980

[28] Escobedo AA, Almirall P, Alfonso M, Cimerman S, Chacín-Bonilla L. Sexual transmission of giardiasis: a neglected route of spread? Acta Trop. 2014;132: 106–111. 10.1016/j.actatropica.2013.12.025 24434784

[29] Narayan S, Galanis E, BC STEI Group. Are enteric infections sexually transmitted in British Columbia? Can Commun Dis Rep. 2016;42: 24–29. 10.14745/ccdr.v42i02a01 29770000 PMC5864255

[30] Mook P, Gardiner D, Kanagarajah S, Kerac M, Hughes G, Field N, et al Use of gender distribution in routine surveillance data to detect potential transmission of gastrointestinal infections among men who have sex with men in England. Epidemiol Infect. 2018;146: 1468–1477. 10.1017/S0950268818001681 29923475 PMC9133680

[31] Pufall EL, Kall M, Shahmanesh M, Nardone A, Gilson R, Delpech V, et al Sexualized drug use (“chemsex”) and high-risk sexual behaviours in HIV-positive men who have sex with men. HIV Med. 2018;19: 261–270. 10.1111/hiv.12574 29368440 PMC5900961

[32] Chang SY, Sun HY, Ji D-D, Lo YC, Wu CH, Wu PY, et al Cost-Effectiveness of Detection of Intestinal Amebiasis by Using Serology and Specific-Amebic-Antigen Assays among Persons with or without Human Immunodeficiency Virus Infection. J Clin Microbiol. 2008;46: 3077–3079. 10.1128/JCM.01151-08 18596142 PMC2546747

[33] Haque R, Huston CD, Hughes M, Houpt E, Petri WA. Amebiasis. N Engl J Med. 2003;348: 1565–1573. 10.1056/NEJMra022710 12700377

[34] Dhanalakshmi S, Meenachi C, Parija SC. Indirect Haemagglutination Test in Comparison with ELISA for Detection of Antibodies against Invasive Amoebiasis. J Clin Diagn Res. 2016;10: DC05–8. 10.7860/JCDR/2016/21566.8326 27656436 PMC5028449

[35] Saidin S, Othman N, Noordin R. Update on laboratory diagnosis of amoebiasis. Eur J Clin Microbiol Infect Dis. 5 ed. European Journal of Clinical Microbiology & Infectious Diseases; 2018;38: 1–24. 10.1007/s10096-018-3411-7 30255429

[36] Abd Alla MD, Wolf R, White GL, Kosanke SD, Cary D, Verweij JJ, et al Efficacy of a Gal-lectin subunit vaccine against experimental Entamoeba histolytica infection and colitis in baboons (Papio sp.). Vaccine. 2012;30: 3068–3075. 10.1016/j.vaccine.2012.02.066 22406457

[37] Quach J, St-Pierre J, Chadee K. The future for vaccine development against Entamoeba histolytica. Hum Vaccin Immunother. 2014;10: 1514–1521. 10.4161/hv.27796 24504133 PMC5396225

