# Supplementary material for: Ongoing transmission of Entamoeba histolytica among newly diagnosed people living with HIV in Taiwan, 2009-2018
Source: PLoS Negl Trop Dis. 2020 Jun 12;14(6):e0008400. doi: 10.1371/journal.pntd.0008400 (PMC7314233; doi:10.1371/journal.pntd.0008400)
Supplement: S1 Table — (PDF) [file pntd.0008400.s001.pdf]

S1 Table. Comparisons of characteristics between newly diagnosed people living with HIV who had indirect hemagglutination assay (IHA) determined at baseline and those who did not

	Total	Tested for IHA	Not tested for IHA	Univariate analysis	Multivariate analysis
	N=5362	N=3499	N=1863	*p*-value	*p*-value
Age, median (IQR), years	29.3 (24.5, 36.0)	28.9 (24.2, 35.5)	29.9 (25.1, 36.9)	**<0.001**	**0.035**
Male, n (%)	5219 (97.3)	3415 (97.6)	1804 (96.8)	0.109	
Mode of transmission, n (%)					
Men who have sex with men	4555 (84.9)	3039 (86.9)	1516 (81.4)	**<0.001**	0.151
People who inject drugs	232 (4.3)	94 (2.7)	138 (7.4)	**<0.001**	**0.001**
Location					
Northern Taiwan	2744 (51.2)	1670 (47.7)	1074 (57.6)	**<0.001**	(ref)
Central Taiwan	796 (14.8)	463 (13.2)	333 (17.9)		**0.020**
Southern Taiwan	1822 (34.0)	1366 (39.0)	456 (24.5)		**<0.001**
Coinfection, n (%)					
HBsAg-positive [N=5258]	527 (10.0)	320 (9.2)	207 (11.5)	**0.010**	0.124
Anti-HCV-positive [N=5296]	418 (7.9)	227 (6.5)	191 (10.5)	**<0.001**	0.917
RPR titer ≥4, n (%) [N=5308]	1063 (20.0)	692 (19.9)	371 (20.3)	0.773	
Anti-HAV IgG-positive, n (%) [N=4601]	795 (17.3)	580 (17.8)	215 (16.1)	0.183	
Enterically transmitted infections, n (%)	124 (2.3)	90 (2.6)	34 (1.8)	0.086	
Shigellosis	4 (0.1)	4 (0.1)	0 (0)	0.305	

Salmonellosis	72 (1.3)	47 (1.3)	25 (1.3)	>0.999	
Giardiasis	14 (0.3)	13 (0.4)	1 (0.1)	**0.044**	0.365
Cryptosporidiosis	11 (0.2)	10 (0.3)	1 (0.1)	0.111	
Acute hepatitis A	28 (0.5)	21 (0.6)	7 (0.4)	0.325	
Any opportunistic infection, n (%)	886 (16.5)	593 (16.9)	293 (15.7)	0.263	
Pneumocystosis	597 (11.1)	406 (11.6)	191 (10.3)	0.145	
Tuberculosis	80 (1.5)	51 (1.5)	29 (1.6)	0.813	
Cryptococcosis	67 (1.2)	48 (1.4)	19 (1.0)	0.303	
White blood cell count, median (IQR), x 10^3 cells/µl [N=5353]	5.6 (4.4, 7.0)	5.6 (4.4, 7.0)	5.7 (4.5, 7.1)	0.226	
Hemoglobin, median (IQR), g/dL [N=5338]	14.1 (12.6, 15.1)	14.0 (12.5, 15.0)	14.2 (12.8, 15.2)	**<0.001**	**0.020**
Any abnormal liver function tests, n (%)	1614 (30.1)	1043 (29.8)	571 (30.6)	0.532	
Plasma HIV RNA load, median (IQR), log_{10} copies/ml [N=5324]	4.8 (4.3, 5.3)	4.8 (4.3, 5.3)	4.8 (4.3, 5.3)	0.281	
HIV RNA load >5 log_{10} copies/ml, n (%)	2154 (40.5)	1405 (40.4)	749 (40.6)	0.907	
CD4 lymphocyte count, median (IQR), cells/µl [N=5355]	289 (133, 441)	285 (127, 437)	296 (144, 448)	0.054	
Presence of diarrhea, n (%)	559 (10.4)	438 (12.5)	121 (6.5)	**<0.001**	**<0.001**

*Boldface indicates a significant result.

†Abbreviations: HAV, hepatitis A virus; HBsAg, hepatitis B virus surface antigen; HCV, hepatitis C virus; IQR, interquartile range; RPR, rapid plasma reagin.
