# Supplementary material for: Ongoing transmission of Entamoeba histolytica among newly diagnosed people living with HIV in Taiwan, 2009-2018
Source: PLoS Negl Trop Dis. 2020 Jun 12;14(6):e0008400. doi: 10.1371/journal.pntd.0008400 (PMC7314233; doi:10.1371/journal.pntd.0008400)
Supplement: S2 Table — (PDF) [file pntd.0008400.s002.pdf]

S2 Table. Comparisons of characteristics between newly diagnosed people living with HIV with and those without high indirect hemagglutination (IHA) titres in univariable analyses

	Total	IHA titre ≥128	IHA titre <128	
	N=3499	N=205	N=3294	*p*-value
Age, median (IQR), years	28.9 (24.2, 35.5)	33.5 (28.8, 40.4)	28.5 (24.0, 35.1)	**<0.001**
Male, n (%)	3415 (97.6)	204 (99.5)	3211 (97.5)	0.061
Mode of transmission, n (%)				
Men who have sex with men	3039 (86.9)	191 (93.2)	2848 (86.5)	**0.004**
People who inject drugs	94 (2.7)	3 (1.5)	91 (2.8)	0.372
Location				
Northern Taiwan	1670 (47.7)	97 (47.3)	1573 (47.8)	0.529
Central Taiwan	463 (13.2)	32 (15.6)	431 (13.1)	
Southern Taiwan	1366 (39.0)	76 (37.1)	1290 (39.2)	
Coinfection, n (%)				
HBsAg-positive [N=3460]	320 (9.2)	32 (15.8)	288 (8.8)	**0.002**
Anti-HCV-positive [N=3485]	227 (6.5)	14 (6.9)	213 (6.5)	0.769
RPR titer ≥4, n (%) [N=3476]	692 (19.9)	71 (35.5)	621 (19.0)	**<0.001**
Anti-HAV IgG-positive, n (%) [N=3264]	580 (17.8)	42 (23.9)	538 (17.4)	**0.033**
Enterically transmitted infections, n (%)	90 (2.6)	10 (4.9)	80 (2.4)	**0.040**
Shigellosis	4 (0.1)	2 (1.0)	2 (0.1)	**0.019**

Salmonellosis	47 (1.3)	5 (2.4)	42 (1.3)	0.195
Giardiasis	13 (0.4)	2 (1.0)	11 (0.3)	0.175
Cryptosporidiosis	10 (0.3)	1 (0.5)	9 (0.3)	0.454
Acute hepatitis A	21 (0.6)	4 (2.0)	17 (0.5)	**0.031**
Any opportunistic infection, n (%)	593 (16.9)	42 (20.5)	551 (16.7)	0.179
Pneumocystosis	406 (11.6)	27 (13.2)	379 (11.5)	0.499
Tuberculosis	51 (1.5)	3 (1.5)	48 (1.5)	>0.999
Cryptococcosis	48 (1.4)	7 (3.4)	41 (1.2)	**0.020**
White blood cell count, median (IQR), x 10^3 cells/µl [N=3497]	5.6 (4.4, 7.0)	5.5 (4.4, 7.4)	5.6 (4.4, 7.0)	0.596
Hemoglobin, median (IQR), g/dL [N=3487]	14.0 (12.5, 15.0)	13.4 (11.5, 14.6)	14.1 (12.6, 15.1)	**<0.001**
Any abnormal liver function tests, n (%)	1043 (29.8)	66 (32.2)	977 (29.7)	0.433
Plasma HIV RNA load, median (IQR), log_{10} copies/ml [N=3478]	4.8 (4.3, 5.3)	4.8 (4.4, 5.2)	4.8 (4.3, 5.4)	0.648
HIV RNA load >5 log_{10} copies/ml, n (%)	1405 (40.4)	79 (38.7)	1326 (40.5)	0.659
CD4 lymphocyte count, median (IQR), cells/µl [N=3497]	285 (127, 437)	267 (109, 393)	286 (128, 438)	0.261
CD4 <200 cells/µl, n (%)	1240 (35.5)	87 (42.4)	1153 (35.0)	0.125
CD4 200-350 cells/µl	954 (27.3)	52 (25.4)	902 (27.4)	
CD4 350-500 cells/µl	655 (18.7)	29 (14.1)	626 (19.0)	
CD4 ≥500 cells/µl	648 (18.5)	37 (18.0)	611 (18.6)	
Presence of diarrhea, n (%)	438 (12.5)	96 (46.8)	342 (10.4)	**<0.001**

*Boldface indicates a significant result.

†Abbreviations: HAV, hepatitis A virus; HBsAg, hepatitis B virus surface antigen; HCV, hepatitis C virus; IQR, interquartile range; RPR, rapid plasma reagin.
