# Supplementary material for: Ongoing transmission of Entamoeba histolytica among newly diagnosed people living with HIV in Taiwan, 2009-2018
Source: PLoS Negl Trop Dis. 2020 Jun 12;14(6):e0008400. doi: 10.1371/journal.pntd.0008400 (PMC7314233; doi:10.1371/journal.pntd.0008400)
Supplement: S3 Table — (PDF) [file pntd.0008400.s003.pdf]

S3 Table. Factors associated with a high indirect hemagglutination (IHA) titre (≥1:128) in multivariable analysis

	Adjusted odds ratio	*p*-value
Age, per 1-year increase	1.003 (1.002-1.004)	<0.001
Men who have sex with men	1.052 (1.026-1.078)	<0.001
Rapid plasma reagin titre ≥1:4	1.048 (1.028-1.068)	<0.001
Shigellosis	1.691 (1.310-2.182)	<0.001
Acute hepatitis A	1.121 (1.013-1.241)	0.027
Cryptococcosis	1.085 (1.011-1.164)	0.024

Variables entered in the multivariable analysis include age, being men who have sex with men, positive hepatitis B surface antigen, rapid plasma reagin titre ≥1:4, positive hepatitis A immunoglobin G, shigellosis, acute hepatitis A, and cryptococcosis.
