# Supplementary material for: Ongoing transmission of Entamoeba histolytica among newly diagnosed people living with HIV in Taiwan, 2009-2018
Source: PLoS Negl Trop Dis. 2020 Jun 12;14(6):e0008400. doi: 10.1371/journal.pntd.0008400 (PMC7314233; doi:10.1371/journal.pntd.0008400)

S1 Fig. Proportion of newly diagnosed people living with HIV who underwent indirect hemagglutination (IHA) testing between 2009 and 2018

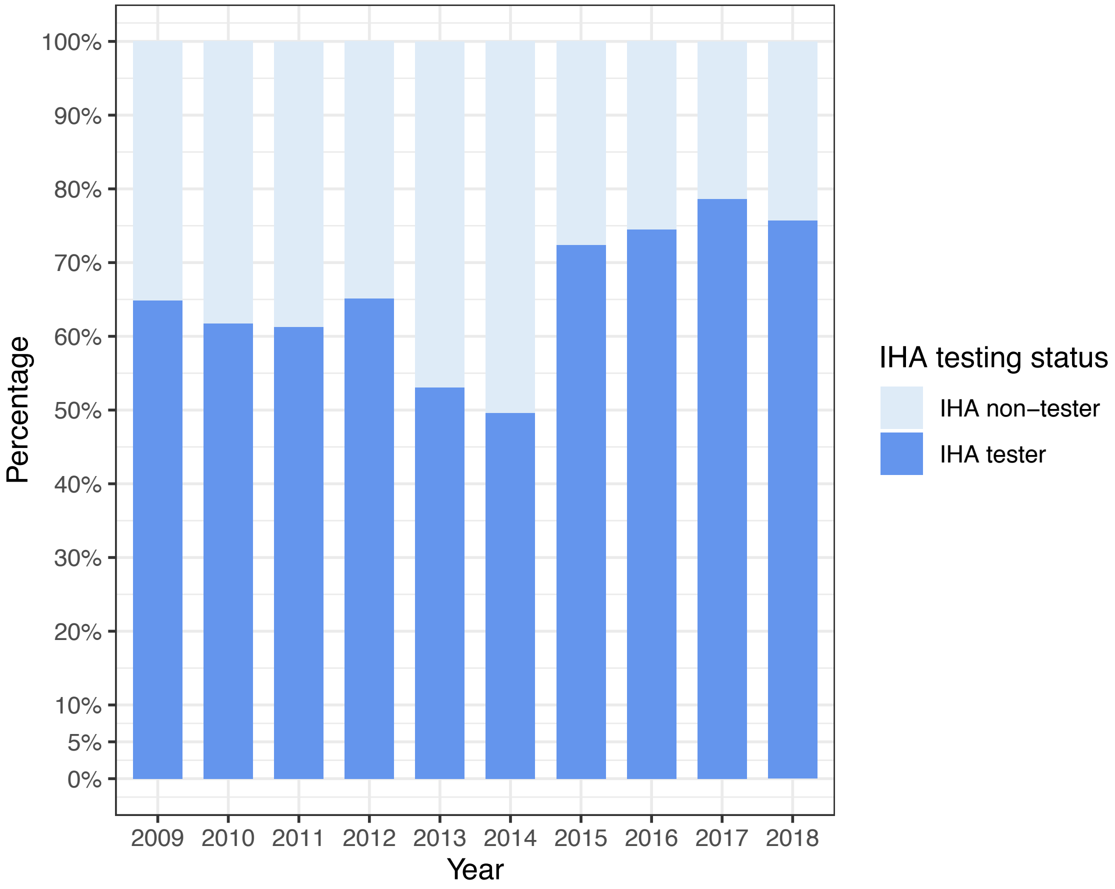

Supplement: S1 Fig — (PDF) [file pntd.0008400.s004.pdf]
