# Supplementary material for: Ongoing transmission of Entamoeba histolytica among newly diagnosed people living with HIV in Taiwan, 2009-2018
Source: PLoS Negl Trop Dis. 2020 Jun 12;14(6):e0008400. doi: 10.1371/journal.pntd.0008400 (PMC7314233; doi:10.1371/journal.pntd.0008400)

S5 Fig. Annual number of indigenous cases of confirmed amoebiasis reported to the Taiwan Centers for Disease Control between 2009 and 2018.

Criteria for confirmed amoebiasis by Taiwan Centers for Disease Control included (1) positive *Entamoeba histolytica* nucleic-acid amplification test from any clinical specimens (including stool, tissue, and aspirate); (2) fever or right upper quadrant pain plus identification of amoebic trophozoites from the tissues; or (3) fever or right upper quadrant pain plus radiographic evidence of liver abscess and positive anti-*E. histolytica* antibody.

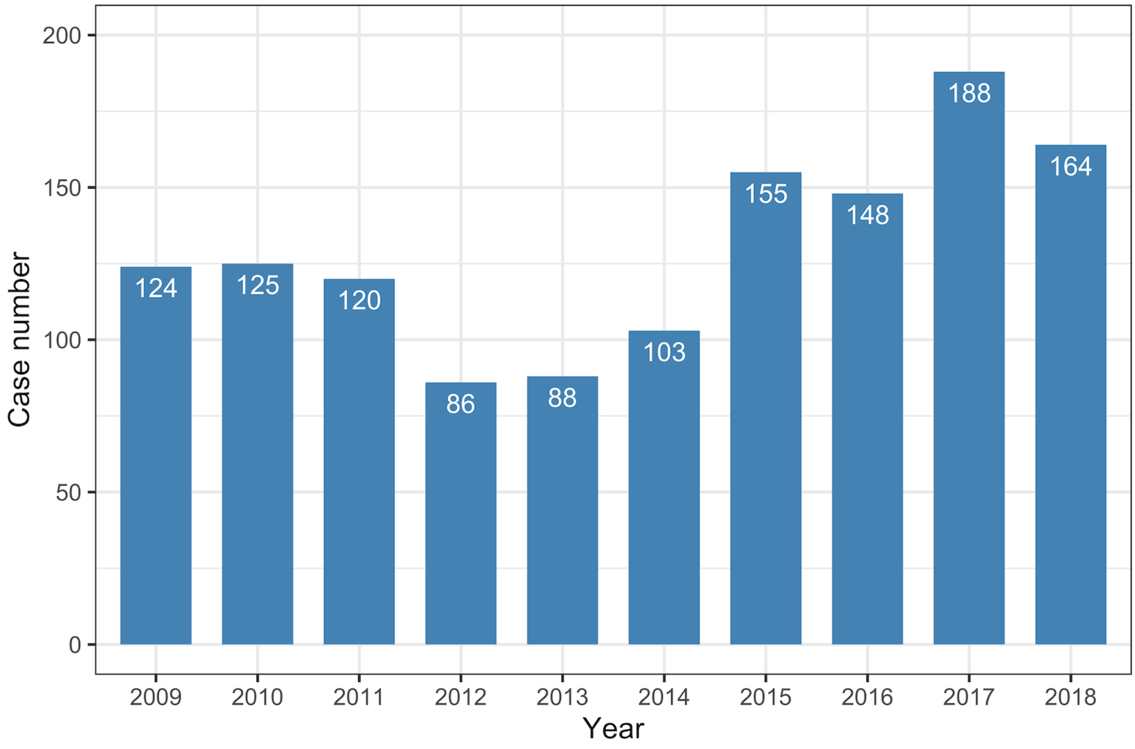

Supplement: S5 Fig — Criteria for confirmed amoebiasis by Taiwan Centers for Disease Control included (1) positive Entamoeba histolytica nucleic-acid amplification test from any clinical specimens (including stool, tissue, and aspirate); (2) fever or right upper quadrant pain plus identification of amoebic trophozoites from the tissues; or (3) fever or right upper quadrant pain plus radiographic evidence of liver abscess and positive anti-E. histolytica antibody. (PDF) [file pntd.0008400.s008.pdf]
